# UMUDGA: A dataset for profiling algorithmically generated domain names in botnet detection

**DOI:** 10.1016/j.dib.2020.105400

**Published:** 2020-03-09

**Authors:** Mattia Zago, Manuel Gil Pérez, Gregorio Martínez Pérez

**Affiliations:** Department of Information Engineering and Communications, University of Murcia, Campus Espinardo Murcia 30100 Spain

**Keywords:** Domain Generation Algorithm (DGA), Natural Language Processing (NLP), Machine learning, Data, Network security

## Abstract

In computer security, botnets still represent a significant cyber threat. Concealing techniques such as the dynamic addressing and the domain generation algorithms (DGAs) require an improved and more effective detection process. To this extent, this data descriptor presents a collection of over 30 million manually-labeled algorithmically generated domain names decorated with a feature set ready-to-use for machine learning (ML) analysis. This proposed dataset has been co-submitted with the research article ”UMUDGA: a dataset for profiling DGA-based botnet” [1], and it aims to enable researchers to move forward the data collection, organization, and pre-processing phases, eventually enabling them to focus on the analysis and the production of ML-powered solutions for network intrusion detection. In this research, we selected 50 among the most notorious malware variants to be as exhaustive as possible. Inhere, each family is available both as a list of domains (generated by executing the malware DGAs in a controlled environment with fixed parameters) and as a collection of features (generated by extracting a combination of statistical and natural language processing metrics).

**Specification table**

**Table:** 

Subject area	Computer Network and Communications, Artificial Intelligence
More specific subject area	Network Security, Machine Learning, Natural Language Processing, Intrusion Detection Systems
Type of data	TXT, CSV, and ARFF files.
How data were acquired	Domain Generation Algorithms have been implemented, executed and their data have been collected and processed to extract the identified features.
Data format	**Raw**: list of Fully Qualified Domain Names (FQDNs) in form of TXT files. **Analyzed**: list of features in form of ARFF and CSV files.
Parameters for data collection	Domain Generation Algorithms (DGAs) have been executed to collect a fixed number of generated domains. Whenever required, the random generator has been initialized with the string “3138C81ED54AD5F8E905555A6623C9C9”.
Description of data collection	**Phase 1**: 37 DGAs have been collected and executed to generate at least 10,000 AGDs. One million legitimate FQDNs have also been added to the collection, for a total of 38+ million domain names. **Phase 2**: Each FQDN has been processed and compared with the English language to extract 100+ numerical features.
Data source location	Faculty of Computer Science, University of Murcia, Murcia, Spain
Data accessibility	**Data repository**: UMUDGA: University of Murcia Domain Generation Algorithm Dataset [2]. Data identification number: 10.17632/76knkx3fzv.1 Direct URL to data: https://data.mendeley.com/datasets/y8ph45msv8/1**Source code repository**: UMUDGA - University of Murcia Domain Generation Algorithm Dataset [3] Source code URL: https://github.com/Cyberdefence-Lab-Murcia/UMUDGA
Related research article	Zago, Mattia and Gil Pérez, Manuel and Martínez Pérez, Gregorio. “UMUDGA: a dataset for profiling DGA-based botnet.” *Computers & Security* (2020): 101719. doi:10.1016/j.cose.2020.101719[1]

## Value of the data

• The proposed dataset aims to overcome the shortage of standard and publicly available data regarding DGA-based malwares. Its value resides in serving as a foundation for benchmarks that eventually might lead to replicable and comparable experiments.
• The primary recipients of the data are the academic scientists that focus on machine-learning-driven network security researches. They might greatly benefit from these freshly generated and carefully reviewed data.
• By shifting the researchers’ attention from the data to the possible solutions, this work aims to ease the development of further experiments, which might eventually lead to innovation in the field of network cybersecurity.
• These data, methods, and code sources are distributed under an open license. We guarantee essential properties such as the comparability and testability of each component.

## 1 Data

**Fig. 1:**
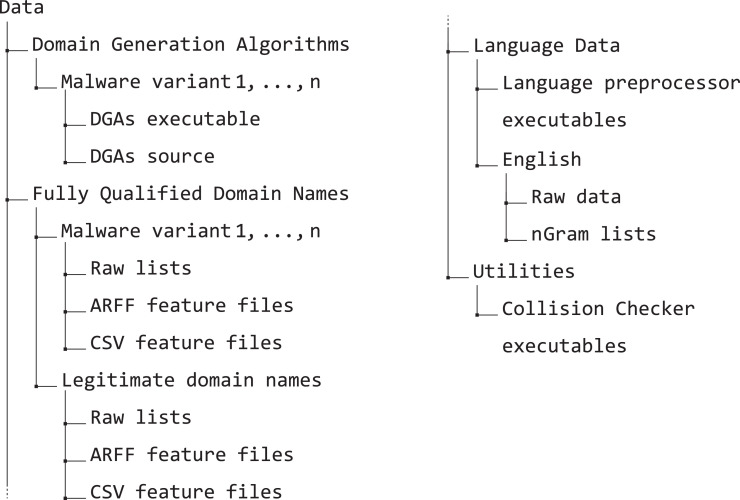
Dataset organization.

The proposed dataset is publicly available through Mendeley Data [2]. As depicted in Fig. 1, the dataset is composed of four root folders that encompass different functionalities and scopes. In order of importance there are:

• *The domain generation algorithms* – in this folder, for each malware variant, there are the DGA executable, the source code, and the reference to the analysis.
• *The actual data folder (named Fully Qualified Domain Names)* – in this folder, for each malware variant plus the legitimate domains, there are three subfolders:
  - *Raw list* – includes the TXT lists of Fully Qualified Domain Names (FQDNs) in different tiers (e.g., 1000, 10,000);
  - *ARFF features* – includes the data processed and exported in the TXT (see [4]) format.
  - *CSV features* – includes the data processed and exported as comma-separated CSV files.
• *The language data* – in this folder, there are the executables to preprocess any given language and the preprocessed, ready-to-use data for the English language (*i.e*., the raw wordlists obtained from the Leipzig Corpora [5] and the lists of extracted *n*Grams).
• *The utility folder* – in this folder, there are the executables and the source codes for any relevant package that might be helpful for the researchers, *e.g*., the collision checker.

In the following sections, we will refer to several figures and tables. Specifically:

• Figures:
  - Dataset structure – the figure mentioned above (Fig. 1) reports the Mendeley Data [2] repository structure;
  - Framework architecture – from the main co-submitted article [1, Fig. 3], describes the implemented architecture and module names.
• Tables:
  - The list of features generated by the NLP Processor for each FQDN (Table 1) – presents the feature code, description, and mathematical definition of each implemented feature. Note that the *n*Grams features are described in Section 2.3.2;

**Table 1:** List of features generated by the *NLP Processor* for each FQDN.

Code	Description	Definition
L-*x*	String length of *x* domain level	$l_{x}=\left|d_{x}\right|$
N	Number of domain levels	n=|PARTS(d)|
LC-C	Longest consecutive consonant sequence	${lc}_{c}=\mathrm{LCS}\left(d,C\right)$
LC-D	Longest consecutive number sequence	${lc}_{d}=\mathrm{LCS}\left(d,D\right)$
LC-V	Longest consecutive vowel sequence	${lc}_{v}=\mathrm{LCS}\left(d,V\right)$
R-CON-*x*	Ratio of consonants characters	$r_{x,c}=R\left(d,C\right)$
R-LET-*x*	Ratio of letter characters	$r_{x,l}=R\left(d,C\cup V\right)$
R-NUM-*x*	Ratio of numerical characters	$r_{x,n}=R\left(d,D\right)$
R-SYM-*x*	Ratio of symbolical characters	$r_{x,s}=R\left(d,S\right)$
R-VOW-*x*	Ratio of vowel characters	$r_{x,v}=R\left(d,V\right)$

where x∈{FQDN,2LD,OLD} denotes the domain levels.

C=b,c,d,f,g,h,j,k,l,m,n,p,q,r,s,t,v,w,x,y,z

V=a,e,i,o,u

D=0,1,2,3,4,5,6,7,8,9

S=“-”,“.”
  - the general feature statistics (Table 2) – presents the mean, standard deviation, minimum, and maximum metrics for each feature and each *n*Grams set.

**Table 2:** General statistics for the features contained in this dataset.

METRIC	MEAN	STD	MIN	MAX	METRIC	MEAN	STD	MIN	MAX
L-2DN	1.42e16	7.01e16	1.00e01	4.50e02	2G-DST-EU	2.75e15	4.13e15	1.69e16	7.45e15
L-FQDN	1.90e16	6.34e15	4.00e01	4.80e02	2G-DST-JI	5.70e16	5.10e15	0	5.21e15
L-ODN	9.00e14	3.39e15	0	2.30e02	2G-DST-KL	2.76e16	1.55e16	0	1.44e15
LC-C	4.79e15	2.90e15	0	3.00e02	2G-DST-MA	1.98e16	2.00e15	1.80e16	2.00e15
LC-V	1.71e16	1.12e16	0	1.90e02	2G-E	3.80e15	2.99e16	2.22e10	2.27e16
N	2.07e16	2.63e15	2.00e01	4.00e01	2G-KEN	1.73e16	2.99e15	6.17e16	2.88e15
R-CON-2DN	6.89e14	1.81e16	0	1.00e01	2G-KUR	9.51e14	3.83e15	2.39e15	1.07e15
R-CON-FQDN	6.32e15	1.27e15	0	9.58e15	2G-MEAN	7.72e15	4.23e00	7.72e15	7.72e15
R-CON-ODN	4.94e15	1.80e15	0	1.00e01	2G-NORM	3.57e16	2.52e16	5.62e08	2.17e15
R-LET-2DN	9.45e15	1.43e16	0	1.00e01	2G-PEA	7.46e15	4.36e15	4.35e15	8.84e15
R-LET-FQDN	8.90e15	1.18e15	1.62e16	9.74e15	2G-PRO	5.51e16	4.77e15	1.12e10	3.91e15
R-LET-ODN	6.95e15	2.47e16	0	1.00e01	2G-PSTD	7.18e16	1.22e16	4.07e15	2.07e16
R-NUM-2DN	5.25e16	1.44e16	0	1.00e01	2G-PVAR	5.31e09	1.83e11	1.66e09	4.28e15
R-NUM-FQDN	4.71e16	1.22e15	0	8.11e15	2G-QMEAN	7.23e15	1.21e16	4.14e15	2.07e15
R-NUM-ODN	5.21e15	3.97e14	0	1.00e01	2G-REP	4.48e15	8.23e15	0	1.20e02
R-SYM-2DN	2.31e16	1.43e15	0	4.00e00	2G-SKE	9.49e15	1.71e16	5.09e16	3.16e16
R-SYM-FQDN	6.34e15	2.34e16	2.08e15	3.33e15	2G-SPE	1.99e16	3.44e15	7.09e14	3.31e15
R-SYM-ODN	7.05e09	9.08e15	0	2.00e00	2G-STD	7.19e15	1.22e15	4.07e15	2.07e16
R-VOW-2DN	2.57e15	1.54e15	0	1.00e01	2G-SUMSQ	1.79e15	7.26e14	2.00e01	7.00e02
R-VOW-FQDN	2.58e15	1.07e16	0	8.33e15	2G-TKUR	5.17e15	2.69e15	9.03e12	1.30e16
R-VOW-ODN	2.00e16	8.54e15	0	1.00e01	2G-TPSTD	5.79e15	3.37e15	3.12e08	1.97e16
1G-25P	3.68e11	8.53e15	0	2.70e16	2G-TPVAR	4.49e09	5.20e08	9.73E-01	3.89e09
1G-50P	1.70e16	6.86e15	0	5.26e15	2G-TSKE	2.11e16	5.64e15	8.98e15	3.60e16
1G-75P	5.36e15	2.08e15	0	1.00e00	2G-TSTD	5.79e15	3.37e15	3.12e07	1.97e15
1G-COV	1.38e16	2.28e16	4.11e15	2.41e16	2G-TSUM	5.23e15	4.25e16	1.12e10	3.05e15
1G-DIST	1.22e16	3.31e15	2.00e01	3.10e02	2G-TSUMSQ	5.85e15	6.80e15	1.26e04	5.11e15
1G-DST-CA	3.17e15	1.81e14	1.95e15	3.60e15	2G-TVAR	4.49e09	5.21e08	9.74E-01	3.89e07
1G-DST-CH	1.56e16	4.69e14	5.09e16	6.66e15	2G-VAR	5.31e10	1.83e10	1.66e11	4.28e16
1G-DST-EM	7.15e15	2.46e16	1.01e16	1.90e15	3G-25P	0	0	0	0
1G-DST-EU	3.34e16	4.50e15	1.61e15	7.86e15	3G-50P	0	0	0	0
1G-DST-JI	1.64e16	5.35e15	9.73e15	4.76e15	3G-75P	0	0	0	0
1G-DST-KL	8.41e15	9.72e15	-7.33e+15	3.42e16	3G-COV	1.68e09	3.27e07	9.39e07	6.16e08
1G-DST-MA	1.44e16	1.56e16	7.10e15	2.00e15	3G-DIST	1.59e14	6.28e15	1.00e01	4.40e02
1G-E	3.28e15	1.23e16	2.88e15	9.55e15	3G-DST-CA	6.39e16	5.57e15	6.37e15	6.41e15
1G-KEN	7.05e15	5.88e15	3.29e15	8.74e15	3G-DST-CH	7.43e15	2.90e16	2.88e16	1.00e01
1G-KUR	2.54e16	2.86e15	-2.12e+15	3.10e15	3G-DST-EM	1.01e15	3.55e16	1.19e16	2.58e15
1G-MEAN	2.78e15	2.75e03	2.78e15	2.78e16	3G-DST-EU	2.69e15	4.82e14	1.58e16	1.00e16
1G-NORM	4.71e15	1.40e15	6.52e15	9.53e14	3G-DST-JI	1.51e15	4.66e15	0	1.65e16
1G-PEA	9.20e15	2.79e14	5.92e15	9.85e14	3G-DST-KL	1.27e16	2.28e15	0	4.86e15
1G-PRO	8.28e14	3.45e15	5.05e15	2.65e16	3G-DST-MA	2.00e16	1.85e16	1.94e16	2.00e15
1G-PSTD	4.56e15	8.32e14	1.53e16	1.21e16	3G-E	5.43e16	7.79e15	0	8.98e15
1G-PVAR	2.15e15	7.88e15	2.33e15	1.47e16	3G-KEN	5.09e14	9.64e14	1.30e15	8.62e15
1G-QMEAN	5.36e16	7.08e15	3.17e15	1.24e16	3G-KUR	3.41e15	1.32e16	1.06e16	4.67e16
1G-REP	4.12e15	2.40e16	0	1.70e02	3G-MEAN	2.14e11	1.71E-02	2.14e11	2.14e10
1G-SKE	1.66e16	5.57e14	-2.81e+16	5.41e15	3G-NORM	4.54e15	6.39e15	0	1.01e15
1G-SPE	8.22e15	5.90e15	3.97e15	9.63e15	3G-PEA	6.25e15	1.72e15	5.93e15	7.89e15
1G-STD	4.62e15	8.44e14	1.55e16	1.23e15	3G-PRO	6.38e15	1.09e16	0	1.64e16
1G-SUMSQ	3.36e15	1.95e14	3.00e01	2.08e03	3G-PSTD	1.23e15	2.27e16	6.98e15	4.63e15
1G-TKUR	4.14e15	2.93e15	-3.73e+15	3.56e15	3G-PVAR	1.55e10	6.00e08	4.87e08	2.14e10
1G-TPSTD	2.81e16	4.52e15	5.29e15	3.50e16	3G-QMEAN	1.23e16	2.27e16	6.98e15	4.63e16
1G-TPVAR	8.13e15	2.42e16	2.80e11	1.22e16	3G-REP	5.06e15	2.89e14	0	1.10e02
1G-TSKE	2.14e15	5.14e15	1.03e16	5.95e16	3G-SKE	5.73e15	1.06e16	3.25e16	2.16e16
1G-TSTD	2.85e16	4.59e15	5.36e15	3.55e15	3G-SPE	5.25e15	9.96e15	1.34e16	8.91e15
1G-TSUM	5.32e15	1.45e15	3.80e15	9.61e15	3G-STD	1.23e16	2.27e16	6.98e15	4.63e15
1G-TSUMSQ	3.77e15	1.27e16	1.07e15	6.45e15	3G-SUMSQ	1.60e16	6.46e15	1.00e01	5.80e02
1G-TVAR	8.36e15	2.48e15	2.88e11	1.26e16	3G-TKUR	3.14e16	1.28e16	3.74e16	4.67e16
1G-VAR	2.21e15	8.10e14	2.40e16	1.51e15	3G-TPSTD	1.55e10	2.50e11	0	1.65e16
2G-25P	0	0	0	0	3G-TPVAR	8.65e05	3.30e07	0	2.71e08
2G-50P	0	0	0	0	3G-TSKE	1.69e16	4.07e15	6.01e15	2.16e16
2G-75P	0	0	0	0	3G-TSTD	1.55e11	2.50e11	0	1.65e16
2G-COV	1.33e10	1.52e10	8.34e09	2.25e10	3G-TSUM	6.19e15	1.00e16	0	1.02e15
2G-DIST	1.64e16	6.04e15	2.00e01	4.50e02	3G-TSUMSQ	4.04e10	1.54e16	0	1.26e16
2G-DST-CA	7.42e15	2.96e15	7.34e15	7.61e15	3G-TVAR	8.65e05	3.30e06	0	2.71e08
2G-DST-CH	8.41e15	3.42e14	2.86e16	6.59e15	3G-VAR	1.55e10	6.00e08	4.87e08	2.14e11
2G-DST-EM	2.66e16	9.27e14	2.73e16	6.69e15
• Algorithms:
  - Algorithm 1 (LCS(d,A))– presents the pseudocode for the Longest Consecutive Sequence algorithm;
  - Algorithm 2 (PE(d,p))– presents the pseudocode for the percentiles calculation algorithm;
  - Algorithm 3 (R(t,A))– presents the pseudocode for the ratio of characters algorithm;

Alongside with the Mendeley Data [2], there is a duplicated copy of the source code, packages, executables, and documentation in a Github public repository [3] that serves as the official project page. Moreover, the Github wiki page “Feature Statistics” [3] also provides metrics and charts for each feature calculated and available in the dataset.

## 2 Experimental design, materials and methods

Before introducing the dataset, it is worth mentioning a few terms and definitions that will be used throughout the article. Firstly, with *botnet* we identify an group of infected machines, called *bots* or *zombies*, that communicates with of one or more of the Command & Control (C&C) servers that act as a relay for the commands issued by the *botmaster* (botnet owner). Bots often use pseudo-random domain generators, called *domain generation algorithms (DGAs)*, to communicate with the C&C servers. These DGAs generate thousands of domain names, called *algorithmically generated domains (AGDs)*. A deep dive on the subject, with specific attention to machine learning (ML) techniques, is offered by Plohmann et al. [6], [7], [8].

The primary research article [1] thoroughly describes the architecture of the data generation framework (see [1, Fig. 3]). To be precise, the figure highlights both the required inputs (the malware DGAs and the English Language Data) and the provided outputs (the AGD lists and the AGD features sets) that have been implemented to guarantee the scientific accuracy and reproducibility of the dataset.

A selected list of 50 malware variants has been collected, analyzed, processed, and included in the proposed dataset to be as complete as possible. The primary research article [1, Table 1] presents these malware variants according to their tier level, *i.e*., the number of AGDs generated for that specific malware variant. It is important to remark that several variants such as *Pizd, Gozi*, or *Rovnix* have wordlist-based DGAs; thus, their possible AGDs are limited.

Firstly, each of the 50 malware variant DGAs included in the dataset has been collected from online sources [9], [10], [11] and implemented in a module named *Domain List Generation*. Their fixed initialization parameters are described in the following dedicated subsection. To be more precise, whenever a malware variant, such as Gozi, needs one or more wordlists in order to generate the domain names, we have considered each wordlist as a separate variant and memorized the wordlist itself in the corresponding DGA folder.

Secondly, the raw lists of AGDs are then processed by the secondary module, named *Feature Extraction*, that calculates the features according to their formal definitions as described in the following dedicated subsection.

The generated AGDs lists present 551 collisions, which are available in a separate file in the root of the project. To be more precise:

• The variant Gozi (Nasa wordlist) shares
  - 14 AGDs with the variant Matsnu
  - 5 AGDs with the variant Gozi (RFC 4343 wordlist)
• The variant Gozi (RFC 4343 wordlist) shares
  - 5 AGDs with the variant Gozi (Nasa wordlist)
  - 1 AGD with the variant Nymaim
  - 24 AGDs with the variant Matsnu
• The variant Matsnu shares
  - 14 AGDs with the variant Gozi (Nasa wordlist)
  - 24 AGDs with the variant Gozi (RFC 4343 wordlist)
  - 53 AGDs with the variant Nymaim
• The variant Nymaim shares
  - 1 AGD with the variant Gozi (RFC 4343 wordlist)
  - 53 AGDs with the variant Matsnu
  - 3 AGDs with the variant Suppobox (1st version)
  - 5 AGDs with the variant Suppobox (2st version)
• The variant Pizd shares
  - 441 AGDs with the variant Suppobox (1st version)
• The variant Proslikefan shares
  - 1 AGD with the variant Simda
  - 1 AGD with the variant Pykspa (noise)
• The variant Pykspa (noise) shares
  - 1 AGD with the variant Proslikefan
  - 3 AGDs with the variant Simda
• The variant Simda shares
  - 1 AGD with the variant Proslikefan
  - 3 AGDs with the variant Pykspa (noise)
• The variant Suppobox (1st version) shares
  - 3 AGDs with the variant Nymaim
  - 441 AGDs with the variant Pizd
• The variant Suppobox (2st version) shares
  - 5 AGDs with the variant Nymaim

### 2.1 Domain list generation

Several independent executables that implement each malware variant DGA constitute the backbone of the *Domain List Generation* module. The main output of this module is a list of AGDs generated by the malware variants, and to be as precise as possible, each DGA implementation utilizes a fixed seed for the pseudorandom number generator (PRNG) and firstly analyzes, whenever available, the original initialization vectors for the specific malware sample analyzed. Each malware family also includes the links fo the source code and the related analysis.

### 2.2 Feature extraction

The *Feature Extraction* module is composed by two independent processes, namely the NLP *Processor* and the *nGrams Processor*. The features extracted are the ones belonging to *Context-Free* family, defined as specified in Def. 1, quoting Zago *et al*. [7]:

Family 1 **Context-Free Feature**

A feature that is related only to a Fully Qualified Domain Name (FQDN) and thus is independent of contextual information, including, but not limited to, timing, origin or any other environment configuration. First and foremost example of this family is the lexical analysis of the domain name.

The *Domain Inspector* processes each AGD generated, as presented in [1, Fig. 3]. To be precise, the two primary submodules mentioned above require validated FQDNs augmented with their *n*Grams sets. Specifically, as reported in [1], this research only focuses on the first three sets of *n*Grams (*i.e*., n=1,2,3).

The first process (*i.e*., the *NLP Processor*) extracts a total of 22 features by analyzing the domain name as a string. Table 1 presents the extracted list with their formal definitions.

The second process (*i.e*., the *nGrams Processor*), compares the different sets of *n*Grams generated by the *Domain Inspector* with the ones provided by the Leipzig Corpora [5] for the English language (one million words from Wikipedia, 2016 update), generating a total of 29 features per *n*Grams. Section 2.3 presents the formal definitions and the algorithms required for extending and validating the feature set.

### 2.3 Feature definitions

In order to provide a formal declaration of the proposed features, it is necessary to establish a set of standard definitions. Firstly, it is necessary to introduce a series of well-defined terms that will be used through most of the definitions. Intuitively, these definitions will refer to the set of *n*Grams (Def. 1) and its distributions, either absolute (Def. 3) or relative (Def. 4), and the formula that calculates it (Def. 2). Moreover, since most of the features aim to compare this distribution with the one obtained from the English language, another series of definitions is necessary, namely the absolute (Def. 6) and relative (Def. 8) distributions and the formulae that calculates them (Def. 5 and Def. 7, respectively). To avoid symbols ambiguity, with | · | we will refer to the size of the collection “ · ”, while with ABS(·) we will refer to the absolute value of the variable “ · ”.

Definition 1 **(*n*Grams Set)**

Let *n* be the length of the *n*Grams. Then we define as *G* the set of all literals (a−z), digits (0−9) and permitted symbols (−) of length *n*. Thus, *G* is represented by the following regular expression: [a-z0-9-]{n}. The set is then lexicographically sorted.

It is important to notice that the Def. 1 explicitly excludes the dot (“.”) character, due to its reserved use as hierarchical separator [12], and the underscore (“_”) character, as per the RFC 1034 [12].

Having the definition of then *n*Grams set, we define the application that transforms any FQDN in a vector of fixed length representing the occurrences of each *n*Grams.

Definition 2 **(*n*Grams Application)**

Let *d* be a FQDN, *G* its sorted *n*Grams set (See Def. 1), *n* the size of the *n*Grams and let *F*(*g, d*) be the absolute frequency for all the *n*Grams *g* ∈ *G* of the domain *d*.

Then we define as *ρ* the linear application that associate each element of *G* of the domain *d* with a real number, in form of a vector of absolute frequencies:

ρ:G→R:∀g∈G,ρ(g)=F(g,d)

Definition 3 **(*n*Grams Vector)**

Let *d* be a FQDN. Then we define as *w_d_* the vector resulting of applying *ρ*( · ) to the *n*Grams set *G* obtained from the domain *d*. Formally:

$$\rho \left(G\right)=w_{d}=\{F\left(g,d\right)|\forall g\in G\}$$

Definition 4 **(*n*Grams Relative Vector)**

Let $w_{d}^{\prime }$ be the vector of relative frequencies obtained by dividing each element of *w_d_* by the total sum. Mathematically:

$$w_{d}^{\prime }=\{\frac{w}{\sum (w_{d})}|w\in w_{d}\}$$

Example 1 Let n=1 and d=google.com. Then *w_d_* has $w_{d}\left[o\right]=3,$ $w_{d}\left[g\right]=2,$ $w_{d}\left[e\right]=w_{d}\left[l\right]=w_{d}\left[c\right]=w_{d}\left[m\right]=1$ and has 0 as result for any other *g* ∈ *G*. It also holds that $w_{d}^{\prime }\left[o\right]=0.33,$ $w_{d}^{\prime }\left[g\right]=0.22,$ $w_{d}^{\prime }\left[e\right]=w_{d}^{\prime }\left[l\right]=w_{d}^{\prime }\left[c\right]=w_{d}^{\prime }\left[m\right]=0.11,$ having 0 for any other element of $w_{d}^{\prime }$.

The obtained *n*Grams vector can be compared with virtually any language data, namely the *n*Grams relative frequency, *i.e*., the frequency of the *n*Grams in the target language.

Definition 5 **(*n*Grams Language Application)**

Let *d* be a FQDN, *G* its sorted *n*Grams set (See Def. 1), *n* the size of the *n*Grams and let *L*(*g, T*) be the absolute frequency in the target language dictionary *T* for all the *n*Grams *g* ∈ *G* of the domain *d*. Within the scope of this article, *T* is the English language dictionary [5].

Then we define as *σ* the linear application that associate each element of *G* of the domain *d* with a real number, in form of a vector of absolute frequencies::

σ:G→R:∀g∈G,σ(g)=L(g,T)

Definition 6 **(*n*Grams Language Vector)**

Let *d* be a FQDN. Then we define as *ϕ_d_* the vector resulting of applying *σ*( · ) to the *n*Grams set *G* obtained from the domain *d*. Formally:

$$\sigma \left(G\right)=\phi_{d}=\{L\left(g,T\right)|\forall g\in G\}$$

Definition 7 **(*n*Grams Language Relative Application)**

Let *d* be a FQDN, *G* its sorted *n*Grams set (See Def. 1), *n* the size of the *n*Grams and let *L*′(*g, T*) be the relative frequency in the target language dictionary *T* for all the *n*Grams *g* ∈ *G* of the domain *d*. Within the scope of this article, *T* is the English language dictionary [5].

Then we define as *σ*′ the linear application that transforms the domain *d* in a vector of relative frequencies:

$$\sigma^{\prime }:G\to R:\forall g\in G,\sigma \left(g\right)=L^{\prime }\left(g,T\right)$$

Definition 8 **(*n*Grams Language Relative Vector)**

Let *d* be a FQDN. Then we define as $\phi_{d}^{\prime }$ the vector resulting of applying *σ*′( · ) to the domain *d*. Formally:

$$\sigma^{\prime }\left(d\right)=\phi_{d}^{\prime }=\{L^{\prime }\left(g,T\right)|\forall g\in G\}$$

Using [5] as source for the English language, the following example holds.

Example 2 Let n=1 and d=google.com.

Then, *ϕ_d_* has $\phi_{d}\left[o\right]=85,719,$ $\phi_{d}\left[g\right]=20,867,$ $\phi_{d}\left[e\right]=140,497,$ $\phi_{d}\left[l\right]=47,521,$ $\phi_{d}\left[c\right]=37,454,$ $\phi_{d}\left[m\right]=27,780$ and has 0 as result for any other *g* ∈ *G*.

Moreover, $\phi_{d}^{\prime }$ has $\phi_{d}^{\prime }\left[o\right]=7.68,$ $\phi_{d}^{\prime }\left[g\right]=2.03,$ $\phi_{d}^{\prime }\left[e\right]=12.02,$ $\phi_{d}^{\prime }\left[l\right]=3.98,$ $\phi_{d}^{\prime }\left[c\right]=2.71,$ $\phi_{d}^{\prime }\left[m\right]=2.61$ with 0 as result for any other *g* ∈ *G*.

#### 2.3.1 Domain name as string

The first set of features are the ones that do not depend on the size of the chosen *n*Grams, and they are presented in Table 1. In the table, we make use of three algorithms: i) the *Longest Consecutive Sequence* (LCS(d,A)), Algorithm 1), that extracts the longest consecutive sequence composed by the elements in the alphabet passed as argument; ii) the *Percentiles calculation* (PE(d,p), Algorithm 2), that calculates the desired percentile from a domain name; and iii) the *Ratio of characters* (R(t,A), Algorithm 3), that calculates the ratios between the tokens contained in the provided alphabet and the target string.

**Algorithm 1:**
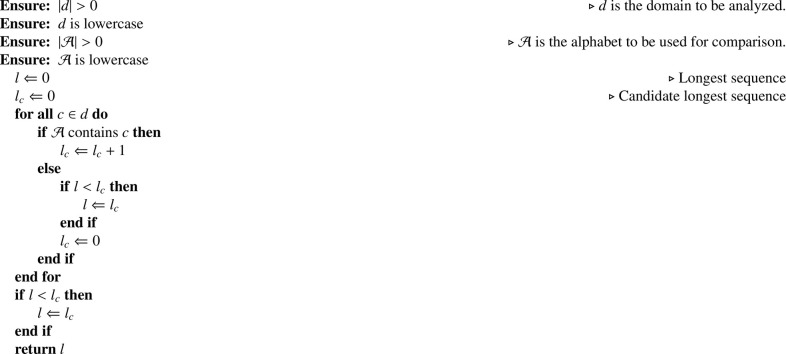
Longest Consecutive Sequence – LCS(d,A).

**Algorithm 2:**
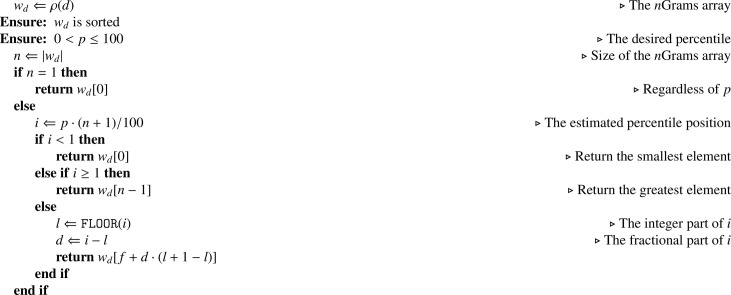
Percentiles calculation – PE(d,p).

**Algorithm 3:**
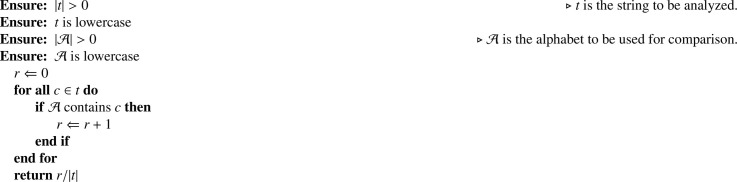
Ratio of characters – R(t,A).

Finally, we indicate with PARTS(d) the list of all the FQDN parts of the domain name, for example, if d=www.um.es, then PARTS(d)=[www,um,es]. These parts are generically called Domain Levels (LD), and in this article we will refer to “es” as the top level domain (TLD), to “um” as the second level domain (2LD) and to “www” concatenated to any other subdomain level as the other level domain (OLD).

The features defined in Table 1 include properties such as the number of domain levels; the longest consecutive sequence of consonants, vowels and numbers; and multiple ratios between set of characters and the domain name.

#### 2.3.2 Domain name as *n*GRAM

With regards to the features that depend on the size of the *n*Grams, the following paragraphs introduce their formal definitions with the relative description and mathematical notation. Each feature is repeated for each distinct value of *n*, in this proposed dataset (available at [2]) the values of *n* are n=1,2,3. In the following paragraphs, each feature is individually formalised.

Feature *n*G-*x*P: Frequencies Percentiles A percentile indicates the value below which a given percentage of observations in a group of observations falls. For each value of *n*, calculates the x={25,50,75} percentile value using Algorithm 2.

Feature *n*G-DIST: Number of distinct *n*Grams Let *d* be a FQDN and *G* its *n*Grams set (See Def. 1). The number of distinct *n*Grams is defined as the size of *G*:

nG-DIST=|G|

Feature *n*G-REP: Number of repeated *n*Grams Let *d* be a FQDN, *G* its *n*Grams set (See Def. 1) and *w_d_* the *n*Grams vector (See Def. 3). The number of repeated *n*Grams is defined as the count of the elements of *w_d_* that are greater than one. Formally:

$$nG\text{-}\mathrm{REP}=\mathrm{COUNT}(w\in w_{d}|w\geq 1)$$

Feature *n*G-E: Entropy Entropy is the average rate at which information is produced by a stochastic source of data.

Mathematically, let $\phi_{d}^{\prime }$ be the English relative vector (See Def. 8) of the domain *d*., then the entropy of the domain is defined as:

$$\mathrm{nG}\text{-}E=-\sum_{\phi \in \phi_{d}^{\prime }}\phi \cdot log_{2}\phi$$

Feature *n*G-COV: Covariance The sample covariance is a measure of the joint variability of two random variables.

Let $w_{d}^{\prime }$ be the *n*Grams relative vector (See Def. 4) and $\phi_{d}^{\prime }$ be the *n*Grams language vector (See Def. 6). Covariance allows us to determine if exists dependence between $w_{d}^{\prime }$ and $\phi_{d}^{\prime }$ by a given *d*. We will use the following formula:

$$nG\text{-}\mathrm{COV}=\frac{1}{|G|-1}\cdot \sum_{i}^{|w_{d}^{\prime }|}\left(w_{d_{i}}^{\prime }-\bar{w_{d}^{\prime }}\right)\left(\phi_{d_{i}}-\bar{\phi_{d}^{\prime }}\right)$$

Where: $\bar{\cdot }$ = arithmetic mean of “ · ”.

Feature *n*G-KEN: Kendall’s Correlation Kendall’s Tau-b rank correlation of the domain *d* with respect to the English language.

Let *i, j* be two independent indexes running from 0 to the size $|w_{d}^{\prime }\left|=\right|\phi_{d}^{\prime }|$ (See Def. 4 and Def. 8). Then, for any two pair $\left(w_{i}\in w_{d}^{\prime },\phi_{i}\in \phi_{d}^{\prime }\right)$ and $(w_{j}\in w_{d}^{\prime },\phi_{j}\in \phi_{d}^{\prime }),$ Kendall’s Correlation defines them as:

• *concordant* if it holds that *w_i_* < *ϕ_j_* and *ϕ_i_* < *ϕ_j_* or *w_j_* < *ϕ_i_* and *ϕ_j_* < *ϕ_i_*;
• *discordant* if it holds that *w_i_* < *w_j_* and *ϕ_j_* < *ϕ_i_* or *w_j_* < *w_i_* and *ϕ_i_* < *ϕ_j_*;
• neither *concordant* nor *discordant* if it holds that $w_{i}=w_{j}$ or $\phi_{i}=\phi_{j}$.

It follows:

$$nG\text{-}\mathrm{KEN}=\frac{n_{c}-n_{d}}{\sqrt{\left(n_{0}-n_{h}\right)\left(n_{0}-n_{k}\right)}}$$

where: $n_{0}=\frac{n(n-1)}{2}$;

*n_c_* = Number of concordant pairs;

*n_d_* = Number of discordant pairs;

*n_k_* = $\sum_{k}\frac{t_{k}\left(t_{k}-1\right)}{2}$;

*n_h_* = $\sum_{h}\frac{u_{h}\left(u_{h}-1\right)}{2}$;

*t_k_* = Number of tied values in the *k^th^* group of ties in $w_{d}^{\prime }$;

*t_h_* = Number of tied values in the *h^th^* group of ties in $\phi_{d}^{\prime }$.

Feature *n*G-PEA: Pearson’s Correlation Computes Pearson’s product-moment correlation coefficients of the domain *d* with respect to the English language.

Let $w_{d}^{\prime }$ be the *n*Grams relative vector (See Def. 4) and $\phi_{d}^{\prime }$ be the *n*Grams language vector (See Def. 8), let also $m=|w_{d}^{\prime }\left|=\right|\phi_{d}^{\prime }|$ be the size of the two vectors. We define as the Pearson’s Correlation the following:

$$nG\text{-}\mathrm{PEA}=\frac{1}{|d|-1}\cdot \frac{1}{\sigma (w_{d}^{\prime })}\cdot \frac{1}{\sigma (\phi_{d}^{\prime })}\cdot \sum_{i}^{m}(w_{d_{i}}^{\prime }-\bar{w_{d}^{\prime }})(\phi_{d_{i}}-\bar{\phi_{d}^{\prime }})$$

where: $\bar{\;\cdot \;}$ = arithmetic mean of “ · ”;

|*d*| = length of the domain name;

*σ*( · ) = standard deviation of “ · ”.

Feature *n*G-SPE: Spearman’s Correlation Computes Spearman’s rank correlation of the domain *d* with respect to the English language. It is implemented with Apache Commons Math SpearmansCorrelation class [13].

Feature *n*G-MEAN: Mean of frequencies Represents the arithmetic mean of the relative frequencies for the domain *d* Mathematically, let $w_{d}^{\prime }$ be the *n*Grams relative vector (See Def. 4) of the domain *d*:

$$nG\text{-}\mathrm{MEAN}=\frac{1}{\left|d\right|}\cdot \sum_{w\in w_{d}^{\prime }}w$$

We will refer to this feature also with the symbol of “$\bar{w}$”.

Feature *n*G-QMEAN: Quadratic mean of frequencies Represents the quadratic mean (or root mean square) of the relative frequencies for the domain *d*. Let $w_{d}^{\prime }$ be the *n*Grams relative vector (See Def. 4) of the domain *d*:

$$nG\text{-}\mathrm{QMEAN}=\sqrt{\frac{1}{\left|d\right|}\cdot \sum_{w\in w_{d}^{\prime }}w^{2}}$$

Feature *n*G-SUMSQ: Squared sum of frequencies Represents the squared sum of the relative frequencies of the domain *d*. Mathematically, let $w_{d}^{\prime }$ be the *n*Grams relative vector (See Def. 4) of the domain *d*:

$$nG\text{-}\mathrm{SUMSQ}=\sum_{w\in w_{d}^{\prime }}w^{2}$$

Feature *n*G-VAR: Variance of frequencies Represents the variance of the relative frequencies of the domain *d* Mathematically, let $w_{d}^{\prime }$ be the *n*Grams relative vector (See Def. 4) of the domain *d*:

$$nG\text{-}\mathrm{VAR}=\frac{1}{|d|-1}\cdot \sum_{w\in w_{d}^{\prime }}{\left(w-\bar{w}\right)}^{2}$$

Feature *n*G-PVAR: Population variance of frequencies Represents the population variance of the relative frequencies of the domain *d*. Mathematically, let $w_{d}^{\prime }$ be the *n*Grams relative vector (See Def. 4) of the domain *d*:

$$nG\text{-}\mathrm{PVAR}=\frac{1}{\left|d\right|}\cdot \sum_{w\in w_{d}^{\prime }}{\left(w-\bar{w}\right)}^{2}$$

Feature *n*G-STD: Standard deviation of frequencies Represents the variance of the relative frequencies of the domain *d*. Mathematically, let $w_{d}^{\prime }$ be the *n*Grams relative vector (See Def. 4) of the domain *d*:

$$nG\text{-}\mathrm{STD}=\sqrt{\frac{1}{|d|-1}\cdot \sum_{w\in w_{d}^{\prime }}{\left(w-\bar{w}\right)}^{2}}$$

Feature *n*G-PSTD: Population standard deviation of frequencies Represents the variance of the relative frequencies of the domain *d*. Mathematically, let $w_{d}^{\prime }$ be the *n*Grams relative vector (See Def. 4) of the domain *d*:

$$nG\text{-}\mathrm{PSTD}=\sqrt{\frac{1}{\left|d\right|}\cdot \sum_{w\in w_{d}^{\prime }}{\left(w-\bar{w}\right)}^{2}}$$

Feature *n*G-KUR: Kurtosis of frequencies Computes the unbiased kurtosis of the relative frequencies of the domain *d*. Let $w_{d}^{\prime }$ be the *n*Grams relative vector (See Def. 4) of the domain *d* and let $m=|w_{d}^{\prime }|$ be its size. It follows:

$$nG\text{-}\mathrm{KUR}=-\frac{3{\left(m-1\right)}^{2}}{\left(m-2)(m-3\right)}+\frac{m(m+1)}{\left(m-1)(m-2)(m-3\right)}\cdot \sum_{w\in w_{d}^{\prime }}(\frac{w-\bar{w}}{\sigma (w_{d}^{\prime })}){}^{4}$$

The kurtosis is not defined for those collections with less than 3 elements. Such event cannot occur in our environment because the size of the vector $|w_{d}^{\prime }|$ is always greater than 3.

Feature *n*G-SKE: Skewness of frequencies Computes the unbiased skewness of the relative frequencies of the domain *d*. Let $w_{d}^{\prime }$ be the *n*Grams relative vector (See Def. 4) of the domain *d* and let $m=|w_{d}^{\prime }|$ be its size. It follows:

$$nG\text{-}\mathrm{SKE}=\frac{m}{\left(m-1)(m-2\right)}\cdot \sum_{w\in w_{d}^{\prime }}(\frac{w-\bar{w}}{\sigma (w_{d}^{\prime })}){}^{3}$$

The skeweness is not defined for those collections with less than 2 elements. Such event cannot occur in our environment because the size of the vector $|w_{d}^{\prime }|$ is always greater than 2.

Feature *n*G-TSUM: Sum of target language frequencies Represents the sum of the English language frequencies for the *n*Grams of *d*. Mathematically, let $\phi_{d}^{\prime }$ be the English relative vector (See Def. 8) of the domain *d*:

$$nG\text{-}\mathrm{TSUM}=\sum_{\phi \in \phi_{d}^{\prime }}\phi$$

Feature *n*G-TSUMSQ: Squared sum of target language frequencies Represents the squared sum of the English language frequencies for the *n*Grams of *d*. Mathematically, let $\phi_{d}^{\prime }$ be the English relative vector (See Def. 8) of the domain *d*:

$$nG\text{-}\mathrm{TSUMSQ}=\sum_{\phi \in \phi_{d}^{\prime }}\phi^{2}$$

Feature *n*G-TVAR: Variance of target language frequencies Represents the variance of the English language frequencies for the *n*Grams of *d*. Mathematically, let $\phi_{d}^{\prime }$ be the English relative vector (See Def. 8) of the domain *d*:

$$nG\text{-}\mathrm{TVAR}=\frac{1}{|d|-1}\cdot \sum_{\phi \in \phi_{d}^{\prime }}{\left(w-\bar{\phi }\right)}^{2}$$

Feature *n*G-TPVAR: Population variance of target language frequencies Represents the population variance of the English language frequencies for the *n*Grams of *d*. Mathematically:

$$nG\text{-}\mathrm{TPVAR}=\frac{1}{\left|d\right|}\cdot \sum_{\phi \in \phi_{d}^{\prime }}{\left(\phi -\bar{\phi }\right)}^{2}$$

Feature *n*G-TSTD: Standard deviation of target language frequencies Represents the variance of the English language frequencies for the *n*Grams of *d*. Mathematically, let $\phi_{d}^{\prime }$ be the English relative vector (See Def. 8) of the domain *d*:

$$nG\text{-}\mathrm{TSTD}=\sqrt{\frac{1}{|d|-1}\cdot \sum_{\phi \in \phi_{d}^{\prime }}{\left(\phi -\bar{\phi }\right)}^{2}}$$

Feature *n*G-TPSTD: Population standard deviation of target language frequencies Represents the variance of the English language frequencies for the *n*Grams of *d*. Mathematically, let $\phi_{d}^{\prime }$ be the English relative vector (See Def. 8) of the domain *d*:

$$nG\text{-}\mathrm{TPSTD}=\sqrt{\frac{1}{\left|d\right|}\cdot \sum_{\phi \in \phi_{d}^{\prime }}{\left(\phi -\bar{\phi }\right)}^{2}}$$

Feature *n*G-TKUR: Kurtosis of target language frequencies Computes the unbiased kurtosis of the English language frequencies for the *n*Grams of *d*. Let $\phi_{d}^{\prime }$ be the English relative vector (See Def. 8) of the domain *d* and let $m=|\phi_{d}^{\prime }|$ be its size. It follows:

$$nG\text{-}\mathrm{TKUR}=-\frac{3{\left(m-1\right)}^{2}}{\left(m-2)(m-3\right)}+\frac{m(m+1)}{\left(m-1)(m-2)(m-3\right)}\cdot \sum_{\phi \in \phi_{d}^{\prime }}(\frac{\phi -\bar{\phi }}{\sigma (\phi_{d}^{\prime })}){}^{4}$$

The kurtosis is not defined for those collections with less than 3 elements. Such event cannot occur in our environment because the size of the vector $|\phi_{d}^{\prime }|$ is always greater than 3.

Feature *n*G-TSKE: Skewness of target language frequencies Computes the unbiased skewness of the English language frequencies for the *n*Grams of *d*. Let $\phi_{d}^{\prime }$ be the English relative vector (See Def. 8) of the domain *d* and let $m=|\phi_{d}^{\prime }|$ be its size. It follows:

$$nG\text{-}\mathrm{TSKE}=\frac{m}{\left(m-1)(m-2\right)}\cdot \sum_{\phi \in \phi_{d}^{\prime }}(\frac{\phi -\bar{\phi }}{\sigma (\phi_{d}^{\prime })}){}^{3}$$

The skeweness is not defined for those collections with less than 2 elements. Such event cannot occur in our environment because the size of the vector $|\phi_{d}^{\prime }|$ is always greater than 2.

Feature *n*G-PRO: Pronounceability Score This feature calculates how pronounceable a domain *d* is, as described by [14, Linguistic Filter 2], it quantifies “the extent to which a string adheres to the phonotactics of the English language”. However, we do consider the whole FQDNs as base for the computation, not only the 2LD.

Let $\phi_{d}^{\prime }$ be the English relative vector (See Def. 8) of the domain *d* and *n* the *n*Grams size. It follows:

$$nG\text{-}\mathrm{PRO}=\frac{\sum (\phi_{d}^{\prime })}{|d|-n+1}$$

Feature *n*G-NORM: Normality Score This feature calculates a score that reflects the attribute of the English language, as defined by [15, Feature 9]. Mathematically, let *w_d_* be the *n*Grams vector (See Def. 3) of the domain *d*, let $\phi_{d}^{\prime }$ be the English relative vector (See Def. 8) of the domain *d*, let $m=|w_{d}\left|=\right|\phi_{d}^{\prime }|$ be their sizes and *n* the *n*Grams size. Thus, the normality score is defined as:

$$nG\text{-}\mathrm{NORM}=\frac{\sum_{i=1}^{m}w_{d_{i}}\cdot \phi_{d_{i}}^{\prime }}{|d|-n+1}$$

Feature *n*G-DST-KL: Kullback-Leiber divergence For a domain *d*, computes the Kullback-Leiber divergence for the vectors $w_{d}^{\prime }$ and $\phi_{d}^{\prime }$. This feature measures how different is $w_{d}^{\prime }$ from $\phi_{d}^{\prime }$. Mathematically, let $w_{d}^{\prime }$ be the *n*Grams relative vector (See Def. 4) of the domain *d*, let $\phi_{d}^{\prime }$ be the English relative vector (See Def. 8) of the domain *d*, let $m=|w_{d}^{\prime }\left|=\right|\phi_{d}^{\prime }|$ be their sizes. The feature is defined as:

$$nG\text{-}\mathrm{DST}\text{-}\mathrm{KL}=\sum_{i}^{m}w_{d_{i}}^{\prime }\ln\left(\frac{w_{d_{i}}^{\prime }}{\phi_{d_{i}}^{\prime }}\right)$$

Feature *n*G-DST-JI: Jaccard Index Measure Computes the Jaccard Index Measure for the vectors $w_{d}^{\prime }$ and $\phi_{d}^{\prime },$ for a given domain *d*. Mathematically, let $w_{d}^{\prime }$ be the *n*Grams relative vector (See Def. 4) of the domain *d*, let $\phi_{d}^{\prime }$ be the English relative vector (See Def. 8) of the domain *d*, let $m=|w_{d}^{\prime }\left|=\right|\phi_{d}^{\prime }|$ be their sizes. The feature is defined as:

$$nG\text{-}\mathrm{DST}\text{-}\mathrm{JI}=1-J(w_{d}^{\prime },\phi_{d}^{\prime }).$$

Where $J(w_{d}^{\prime },\phi_{d}^{\prime })$ is the Jaccard similarity coefficient given by the following expression:

$$J\left(w_{d}^{\prime },\phi_{d}^{\prime }\right)=\frac{\sum_{i=1}^{m}\min\left(w_{d_{i}}^{\prime },\phi_{d_{i}}^{\prime }\right)}{\sum_{i=1}^{m}\max\left(w_{d_{i}}^{\prime },\phi_{d_{i}}^{\prime }\right)}$$

Feature *n*G-DST-CA: Canberra Distance Computes the Canberra Distance. Mathematically, let $w_{d}^{\prime }$ be the *n*Grams relative vector (See Def. 4) of the domain *d*, let $\phi_{d}^{\prime }$ be the English relative vector (See Def. 8) of the domain *d*, let $m=|w_{d}^{\prime }\left|=\right|\phi_{d}^{\prime }|$ be their sizes. The feature is defined as:

$$nG\text{-}\mathrm{DST}\text{-}\mathrm{CA}=\sum_{i=1}^{m}\frac{\mathrm{ABS}(w_{d_{i}}^{\prime }-\phi_{d_{i}}^{\prime })}{\mathrm{ABS}\left(w_{d_{i}}^{\prime }\right)+\mathrm{ABS}\left(\phi_{d_{i}}^{\prime }\right)}$$

where: ABS(·) = absolute value of “ · ”.

Feature *n*G-DST-CH: Chebyshev Distance Computes the Chebyshev Distance between the domain *d* and the English language. Mathematically, let $w_{d}^{\prime }$ be the *n*Grams relative vector (See Def. 4) of the domain *d*, let $\phi_{d}^{\prime }$ be the English relative vector (See Def. 8) of the domain *d*, let $m=|w_{d}^{\prime }\left|=\right|\phi_{d}^{\prime }|$ be their sizes. The feature is defined as:

$$nG\text{-}\mathrm{DST}\text{-}\mathrm{CH}=\max_{\forall i\leq m}\left(\mathrm{ABS}\left(w_{d_{i}}^{\prime }-\phi_{d_{i}}\right)\right)$$

where: ABS(·) = absolute value of “ · ”.

Feature *n*G-DST-EM: Earth Movers Distance Calculates the Earth Movers distance (also known as 1^st^ Wasserstein distance) of the relative frequencies $w_{d}^{\prime }$ with respect to the English language. It is implemented with Apache Commons Math EarthMoversDistance class [13].

Feature *n*G-DST-EU: Euclidean Distance Computes the Euclidean Distance. Mathematically, let $w_{d}^{\prime }$ be the *n*Grams relative vector (See Def. 4) of the domain *d*, let $\phi_{d}^{\prime }$ be the English relative vector (See Def. 8) of the domain *d*, let $m=|w_{d}^{\prime }\left|=\right|\phi_{d}^{\prime }|$ be their sizes. The feature is defined as:

$$nG\text{-}\mathrm{DST}\text{-}\mathrm{EU}=\sqrt{\sum_{i=1}^{m}{\left(w_{d_{i}}^{\prime }-\phi_{d_{i}}^{\prime }\right)}^{2}}$$

Feature *n*G-DST-MA: Manhattan Distance Computes the Manhattan Distance. Mathematically, let $w_{d}^{\prime }$ be the *n*Grams relative vector (See Def. 4) of the domain *d*, let $\phi_{d}^{\prime }$ be the English relative vector (See Def. 8) of the domain *d*, let $m=|w_{d}^{\prime }\left|=\right|\phi_{d}^{\prime }|$ be their sizes. The feature is defined as:

$$nG\text{-}\mathrm{DST}\text{-}\mathrm{MA}=\sum_{i=1}^{m}\mathrm{ABS}\left(w_{d_{i}}^{\prime }-\phi_{d_{i}}^{\prime }\right)$$

where: ABS(·) = absolute value of “ · ”.

#### 2.3.3 Feature Statistics

Table 2 presents classic statistical measures for the features, considering the whole dataset altogether. It is worth mentioning that, for each feature, the class-wise boxplot distribution is available at [3].

By looking at Table 2, it is worth noticing a few values that stand out for two different reasons, namely having a zero value for either the minimum value or the standard deviation one:

• *Having a minimum value equal to zero* – The reason behind these values are to be searched in the nature of the feature. For example, the NLP-1G-MED feature reports the median value of the frequency distribution, which in most of AGDs is zero. However, when considering the NLP-3G-E feature, the reason is quite different. That is, if each 3Gram have zero probability, e.g. the AGD “dajsrmdwhv.tv” belonging to the Kraken (2nd version) variant, then the entropy is defined as zero.
• *Having standard deviation value equal to zero* – In order to have zero standard deviation, all the values of the features must be equals. This is the case of a group of feature calculated over 2Grams and 3Grams, namely NLP-*n*G-25P, NLP-*n*G-50P, NLP-*n*G-75P and NLP-*n*G-MED, where n=2,3. Once again, having most of the terms at zero in the AGDs distributions, cause these features to have themselves a zero value. However, it is not the case for the 1Gram case because of the non-zero probability of each term. However, for completeness, these features are still included in the dataset.

### 2.4 Code and data availability

As specified in the previous section, there are two main code components that interact to generate the proposed dataset, namely the *Domain List Generation* and the *Feature Extraction* modules. The dataset with the released code has been published on the well-known platform Mendeley Data [2]. Fig. 1 highlights the structure of the repository.

#### 2.4.1 Domain list generation module

This module is mainly realized in Python 2.7 and it has been released under the MIT license.

As specified before, the PRNGs have been initialized with a specific seed (either integer or string), available within each DGA source code.

Specifically, the fixed parameters for each DGA are:

• PRNG Seed – Each random generator has been initialized with the hardcoded integer value “521496385”.
• String Seed – Whenever a DGA requires a string seed as initialization vector, the module uses the string:
  “3138C81ED54AD5F8E905555A6623C9C9”.
• Malware variant specific seeds – Security vendors often release, along with the relative signatures, also the initialization vectors for each variant discovered in the wild (either TLDs, numbers, strings, or wordlists). In such cases, the initialization vectors are coded in the generator and marked with online source for reference.
• Random date range – Most of the DGAs require a random date in order to generate the AGDs. When not fixed by some internal constraint, the dates are generated randomly from 01/01/1970 01:00 AM to 01/01/3000 01:10 AM.

#### 2.4.2 Feature extraction module

This module implements the feature definitions as described in Section 2.3. It has been realised in Java 1.8 making use primarily of Apache Commons Math [13] as main library for statistical and mathematical purposes.

The code, however, is closed source and is not, and will not released to the general public.

#### 2.4.3 Technical validation

When considering the list of FQDNs that we assume legitimate, two main problems are to be considered. As specified before, each domain is firstly validated by the Apache Domain Validator library. A total of 178 FQDNs fail to pass the validation procedure. To be more precise:

• 38 of them use one of the new generic top level domains (gTLDs) which are still not included in the list of accepted gTLDs as per the last update of the library (v1.6, 04/02/2017). Namely, .africa (delegated on 14/02/2017), .charity (04/06/2018), .hotels (03/04/2017), .inc (16/07/2018), .sport (08/01/2018);
• 140 domains are technically invalid because of the presence of at least one underscore character (“_”): the validation library checks the domains against the RFC 1123 [16], which limits host names to letters, digits and hyphen. The policy for the underscore character has been clarified later with the RFC 2181 [17, Section 11];
